# Validation of the European SCORE2 algorithm in one European and one Latin-American cohort studies with contrasting populations

**DOI:** 10.1093/ehjqcco/qcag009

**Published:** 2026-01-27

**Authors:** Ko Ko Maung, Eirini Trichia, Henrique Barros, Vlada Hanchar, Jesus Alegre-Díaz, Jaime Berumen, Pablo Kuri-Morales, Roberto Tapia-Conyer, Louisa Gnatiuc Friedrichs, Pedro Marques-Vidal

**Affiliations:** Department of Medicine, Internal Medicine, Lausanne University Hospital (CHUV), University of Lausanne, Lausanne, Switzerland; Clinical Trial Service Unit and Epidemiological Studies Unit, Nuffield Department of Population Health, University of Oxford, Oxford, UK; Epidemiology Research Unit (EPIUnit), Instituto de Saúde Pública da Universidade do Porto (ISPUP), Porto, Portugal; Laboratório para a Investigação Integrativa e Translacional em Saúde Populacional (ITR), Instituto de Saúde Pública da Universidade do Porto (ISPUP), Porto, Portugal; Departamento de Ciências da Saúde Pública e Forenses e Educação Médica, Faculdade de Medicina, Universidade do Porto (FMUP), Porto, Portugal; Epidemiology Research Unit (EPIUnit), Instituto de Saúde Pública da Universidade do Porto (ISPUP), Porto, Portugal; Laboratório para a Investigação Integrativa e Translacional em Saúde Populacional (ITR), Instituto de Saúde Pública da Universidade do Porto (ISPUP), Porto, Portugal; Experimental Research Unit from the Faculty of Medicine, National Autonomous University of Mexico, Mexico City, Mexico; School of Medicine, National Autonomous University of Mexico, Mexico City, Mexico; Experimental Research Unit from the Faculty of Medicine, National Autonomous University of Mexico, Mexico City, Mexico; School of Medicine, National Autonomous University of Mexico, Mexico City, Mexico; Escuela de Medicina y Ciencias de la Salud, Instituto Tecnológico y de Estudios Superiores de Monterrey, Monterrey, Mexico; Experimental Research Unit from the Faculty of Medicine, National Autonomous University of Mexico, Mexico City, Mexico; School of Medicine, National Autonomous University of Mexico, Mexico City, Mexico; Clinical Trial Service Unit and Epidemiological Studies Unit, Nuffield Department of Population Health, University of Oxford, Oxford, UK; Department of Medicine, Internal Medicine, Lausanne University Hospital (CHUV), University of Lausanne, Lausanne, Switzerland

**Keywords:** Primary prevention, Risk assessment, Scores, Validation, Mexico, Portugal

## Abstract

**Aims:**

SCORE2 estimates the 10-year risk of developing non-fatal and fatal cardiovascular disease (CVD), and it was designed for mostly Europeans. However, its transferability to non-European populations has not been extensively studied. The aim of this study was to evaluate the performance of the SCORE2 in ethnically and socioeconomically diverse populations, including those outside of Europe.

**Methods and results:**

We leveraged cohort studies from Mexico (*N* = 95,660, mean age 52.4 years, 32.6% men, mean follow-up 9.7 years), and Portugal (*N* = 1,216, 52.9 years, 36.9% men, 8.8 years follow-up). SCORE2 has differentially classified frequency of CVD events across levels of risk severity, ranging from 0.7–1.0% CVD events among those with low-moderate risk-profiles, vs. 6.6–9.0% among those with high risk-profiles, respectively. The area under the receiver operating curve (AROC) was 0.762 in Mexico and 0.718 in Portugal. Sensitivities (95% CI) were 85.7 (84.2–87.2) and 95.0 (86.1–99.0), and specificities were 42.8 (42.5–43.1) and 24.3 (21.9–26.9) for Mexico and Portugal, respectively. In all cases, the positive predictive values were below 7%, whereas the negative predictive values were all above 98%. Harrell’s C were 0.717 and 0.692, and Brier’s scores were 0.022 and 0.052 for Mexico and Portugal, respectively. In Mexico, SCORE2 showed good calibration, with a slight overestimation in the higher-risk categories. In Portugal, overestimation in higher-risk categories was more substantial.

**Conclusion:**

SCORE2 estimates modest to acceptable performance for predicting the 10-year CVD in Mexicans but performed less favourably in the Portuguese cohort, indicating a need for recalibration.

## Graphical Abstract

**Graphical Abstract:**
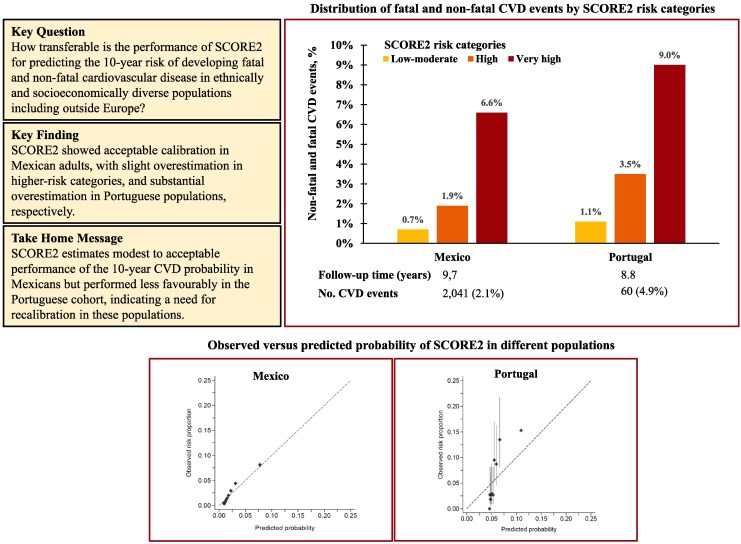

Key Learning Points What is already known:

- SCORE2 is a contemporary cardiovascular risk assessment tool developed to estimate the 10-year risk probability of having a cardiovascular event in contemporary European adults from the general population, based on age, sex, smoking habits, systolic blood pressure (SBP), and high-density or low-density cholesterol levels.
- However, the applicability of SCORE2 in non-European populations with mixed ancestry and potentially different CVD risk profiles has yet to be studied.

What this study adds:

- SCORE2 showed acceptable calibration in Mexican adults, with slight overestimation in higher-risk categories, and substantial overestimation in Portuguese populations, respectively.
- SCORE2 estimates modest to acceptable performance of the 10-year CVD probability in Mexicans but performed less favourably in the Portuguese cohort, indicating a need for recalibration in these populations.

## Introduction

Cardiovascular diseases (CVD) are a leading cause of disability and mortality worldwide. In 2019, CVDs were responsible for almost 19 million deaths, of which nearly 6.5 million occurred prematurely, before the age of 70 years.^1^ Furthermore, it is estimated that 620 million people worldwide are currently living with CVDs.^2^ Given the global increase in the ageing population, together with the increase in multimorbidity and improved survival rates from major CVD events such as ischaemic heart attack and stroke, the burden of CVD is anticipated to increase, particularly in non-Western populations.^3^ Furthermore, the economic burden associated with CVD has increased, with a total of €210 billion annual expenditure in European countries.^4^ Therefore, the need for public health preventive strategies to reduce the excess need for healthcare and the economic burden associated with CVD becomes more pronounced.

CVD prevention includes primary, secondary, and tertiary strategies. Primary prevention aims to prevent CVD events in high-risk individuals through lifestyle changes or pharmacotherapies introduced before disease development. On the other hand, secondary and tertiary prevention focus on preventing further events or improving the quality of life after the disease has been established. Consequently, secondary and tertiary prevention are costly and invasive, and the rate of recurrent CVD events remains high despite adherence to clinical guidelines.^5,6^ Therefore, primary prevention should be prioritized, as it is more cost-effective, and could further prevent premature fatalities through optimal management of pre-existing increases in CVD risk. In this context, the European Society of Cardiology (ESC) recommends an updated CVD risk prediction calculator, known as the Systematic Coronary Risk Evaluation model 2 (SCORE2) algorithm, to assist medical professionals in reliably estimating subsequent cardiovascular risk and to optimally manage people considered high or very high risk, early.^7^

SCORE2 is a contemporary risk assessment tool that estimates the 10-year risk of developing a cardiovascular event, fatal or non-fatal, based on age, sex, smoking habits, systolic blood pressure (SBP), and high or low-density cholesterol levels. The calculator ultimately classifies adults with different levels of CVD risk severity, according to which preventive or pharmacological strategies can be initiated to prevent premature CVD events. The relevance of the SCORE2 algorithm to other populations has been validated in many countries; however, it has not yet been validated in some European countries, such as Portugal. Moreover, the applicability of SCORE2 in non-European populations with mixed ancestry and potentially different CVD risk profiles has yet to be extensively studied. Genetic variations, lifestyle factors, and disease prevalence can differ significantly between European and non-European populations, potentially affecting the performance of SCORE2 in such scenarios.

We aimed to evaluate and compare the performance of the SCORE2 algorithm in two ethnically and socioeconomically diverse populations from and outside Europe.

## Methods

### Study populations

Data from two contemporary population cohorts were leveraged: one Central American (the Mexico City Prospective Study in Mexico) and one European (EpiPorto in Portugal). The details of each cohort are summarized in Supplementary material online, *Table S1.1*. In both cohorts, participants who opted out were followed till the last examination or contact date.

### Recruitment frameworks

The Mexico City Prospective study was initiated in the mid-1990s to assess associations of established and novel risk factors for the main causes of death in Mexico City. Between April 1998 and September 2004, study teams consisting of trained nurses surveyed 159 546 adults aged at least 35 years (67% were women) from two contrasting districts of Mexico City. The two districts were selected for their diverse but settled long-term residents and recent migrants from throughout Mexico, representing people from a wide range of different socio-demographic backgrounds [3]. One district (Coyoacán) has a full spectrum of incomes and tends to be more affluent, while the other district (Iztapalapa) is characterized mostly by middle and lower family incomes. During the home visit, a baseline questionnaire was completed by means of an interviewer-administered electronic handheld data recorder, physical measurements (including resting blood pressure and anthropometry) were taken, and a blood sample was collected for biochemical assaying.^8^

The EPIPorto study is a contemporaneous population-based cohort study,^9^ including adults aged 18 years or older, aiming at assessing the determinants of health among adults from Porto, Portugal. Data from the baseline survey was collected between 1999 and 2003, during which a random sample of 2485 individuals was selected for ongoing evaluations over subsequent periods, resulting in a median follow-up of 7.8 [IQR 7.2–8.5] years.^10^

### Inclusion criteria

Participants were eligible for analysis if they were aged between 40 and 70 years, aligned with the criteria of SCORE2, and its intended use in individuals aged 40 to 69 years.^7^ In addition, only subjects with complete information of interest were included, specifically, smoking status, history of diabetes, history of hypertension, systolic blood pressure (SBP) values, and complete laboratory values for total cholesterol, high-density lipoprotein cholesterol, low-density lipoprotein cholesterol, triglycerides, and eGFR-creatinine (estimated by the Chronic Kidney Disease Epidemiology Collaboration equation,^11^) and follow-up data. We excluded participants with a history of CVD at baseline.

### Cardiovascular outcomes

We defined CVD events that occurred within the first 10 years after recruitment (suggestive of ‘incident’ non-fatal or fatal cases) as an episode of (a) acute or chronic myocardial infarction, (b) symptomatic coronary artery disease, and (c) fatal and non-fatal ischaemic stroke.

### Statistical analysis

First, to calculate and validate SCORE2, the variables of interest (see Supplementary material online, *Tables S1.1* and *S1.2*) were formatted for each cohort according to a standard protocol. Then, a standardized statistical code provided by SCORE2 (score2risk Stata package) and further adapted for the purpose of this study was applied, with all analyses performed onsite (to avoid data security and ethical concerns related to transfer of data). Non-editable, fully anonymised summary statistics files and Kaplan–Mayer survival graphs were collated and sent to the coordination centre.

Participants were initially stratified into low-, high-, and very high-CVD risk categories as defined by the SCORE2 group. The CVD risk categories vary according to age, from low to moderate CVD risk if the estimated SCORE2 was <2.5% at ages <50 years, and <5% at ages 50–69 years. The corresponding age-specific CVD risk was considered high if the estimated SCORE2 was between 2.4% and 7.5%, and between 4.9% and 10%, respectively, and very high if the estimated SCORE2 was ≥7.5% and ≥10%for those aged <50 years old, and 50 to 69 years old, respectively. Kaplan–Meier survival graphs comparing the three risk groups were subsequently computed. Participants were re-classified as low-to-middle and high-to-very-high CVD risk, to subsequently compute sensitivity, specificity, and positive and negative predictive values using accrued CVD events as a reference. The area under the receiver operating characteristic (AROC) curve was computed. The concordance between the predicted and the observed CVD risks was assessed using Harrell’s C-statistic for discrimination. The Brier score was used to assess the overall model performance. The model calibration was assessed in each cohort separately, using the Hosmer–Lemeshow test after splitting the computed SCORE2 risk into deciles or risk and visually presented as calibration plots.

Analyses were performed using Stata version 18 (Stata Corp., College Station, TX, USA).

### Ethical approvals

Both cohorts were approved by the ethical review board of the host institution: The Mexican Ministry of Health, the Mexican National Council for Science and Technology, and the University of Oxford for the Mexico City Prospective Study in Mexico, and EpiPorto CES-320/2016 in Portugal. Written informed consent was obtained from all participants.

## Results

### General characteristics of the participants at baseline

The general characteristics of the participants in each cohort at baseline assessment are summarized in *Table 1*. Overall, the number of participants included in the analysis was 95 660 in Mexico and 1216 in Portugal. Compared to Mexico, Portugal had a higher proportion of men and older participants, although both cohorts had a similar mean baseline age of approximately 52 years. The baseline profile of the CVD-specific risk factors also differed between the two cohorts. The mean systolic blood pressure and the prevalence of current smoking were higher in Portugal than in Mexico (137 mm Hg vs. 128 mm Hg and 43.9% vs. 27.9%, respectively). Likewise, lipid levels were higher in Portugal (average 5.8 mmol/L) than in Mexico (average 4.3 mmol/L), and it was largely driven by differences in LDL-c (2.5 ± 0.8 in Mexico vs. 3.7 ± 1.0 mmol/L in Portugal). By contrast, the prevalence of diabetes and the eGFR-creatinine levels (mL/min/1.73 m^2^) were higher in Mexico than in Portugal (19.7% vs. 5.2% and 98.1 vs. 76.2, respectively).

**Table 1:** Baseline characteristics of the participants included in the analysis stratified by cohort

	Mexico	Portugal
Total participants (*N*)	95 660	1216
Follow-up time (years)	9.7	8.8
Age (years, SD)	52.4 ± 8.3	52.9 ± 8.5
Men (%)	31 144 (32.6)	449 (36.9)
Current smokers (%)	26 738 (27.9)	534 (43.9)
Systolic blood pressure (mmHg)	127.9 ± 16.1	137 ± 37.6
Lipids (mmol/L)		
Total cholesterol	4.3 ± 1.0	5.8 ± 1.1
HDL cholesterol	1.0 ± 0.2	1.5 ± 0.4
LDL cholesterol	2.5 ± 0.8	3.7 ± 1.0
Triglycerides	1.6 ± 0.6	1.4 ± 0.9
eGFR (CKD-EPI) (mL/min/1.73 m^2^)	98.1 ± 13.9	76.2 ± 17.3
Diabetes mellitus (%)	18 887 (19.7)	63 (5.2)

HDL, high-density lipoprotein; LDL, low-density lipoprotein; eGFR, estimated glomerular filtration rate; CKD-EPI, Chronic Kidney Disease Epidemiology Collaboration equation.

The results are expressed as the number of participants (%) for categorical variables or as mean ± standard deviation (SD) for continuous variables.

### Distribution of CVD events

**Figure 1:**
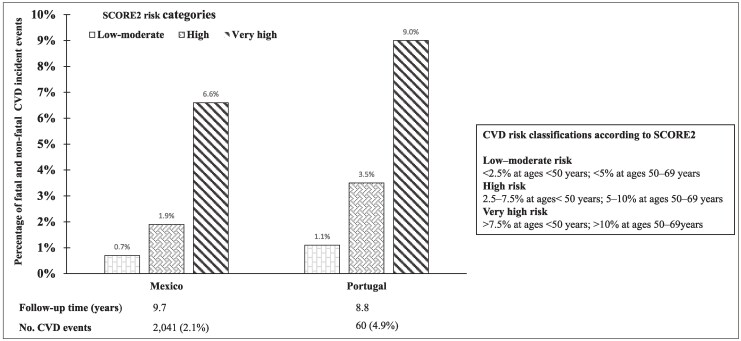
Distribution of fatal and non-fatal CVD events according to SCORE2 risk categories in the cohort populations.

The mean 10-year follow-up periods of the two population cohorts were 9.7 years in Mexico (as censored from the entire follow-up time) and 8.8 years in Portugal. During the follow-up period, a total of 2041 (2.13%) participants from Mexico and 60 (4.9%) participants from Portugal developed major CVD events within the first 10 years of FU, respectively. When SCORE2-risk levels were applied, the crude prevalence of CVD events was 0.7% (Mexico) and 1.1% (Portugal) among participants within the low-to-moderate baseline risk category, 1.9% (Mexico) and 3.5% (Portugal) among participants within the high-risk baseline category, and 6.6% (Mexico) and 9.0% (Portugal) among participants within the very high-risk category (*Table 2*, *Figure 1*).

**Table 2:** Distribution of fatal and non-fatal cardiovascular events according to SCORE2 risk categories, stratified by cohort

CVD risk severity	Mexico		Portugal	
	No event	CVD Event	No event	CVD Event
Low-moderate	40 045 (99.3)	291 (0.7)	281 (98.9)	3 (1.1)
High	39 950 (98.1)	787 (1.9)	468 (96.5)	17 (3.5)
Very high	13 624 (93.4)	963 (6.6)	407 (91.1)	40 (9.0)

Participants were classified into 3 categories according to the predicted 10-year risk of major coronary events according to the SCORE2 algorithm as follows: Low–moderate risk (<2.5% at ages <50 years; <5% at ages 50–69 years); High risk (2.5–7.5% at ages< 50 years; 5–10% at ages 50–69 years); Very high risk (>7.5% at ages <50 years; >10% at ages 50–69 years).

**Figure 2:**
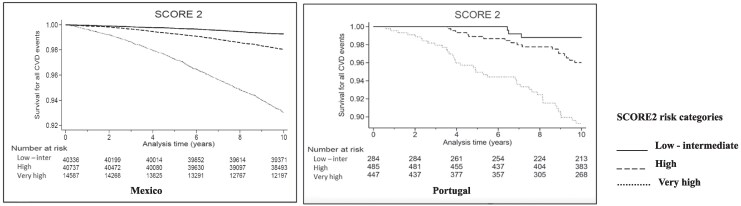
Cohort-specific differences in Kaplan-Meier survival curves given differences in SCORE2 CVD risk categories.

Kaplan-Meier survival curves according to risk categories are provided in *Figure 2*. The low CVD risk group in all cohorts maintained a substantially higher event-free survival probability than the high CVD risk groups. Event-free survival probabilities differed considerably between the high and very high CVD risk groups.

### Performance of the SCORE2 algorithm

**Figure 3:**
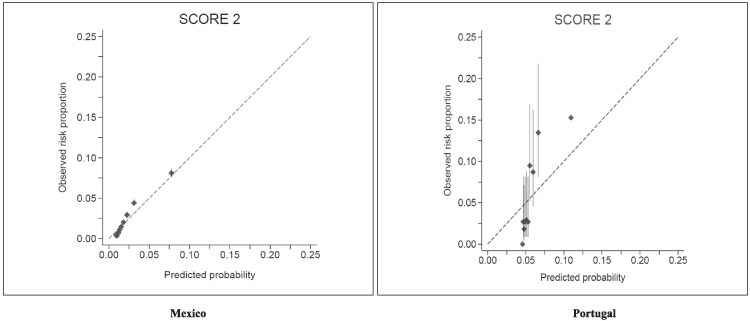
SCORE2 estimated area under receiver operating curve (AROC) for each cohort population.

The diagnostic capacity, concordance, and calibration of the SCORE2 algorithm for all CVD events (fatal and nonfatal) across the cohorts are summarized in *Table 3*. The SCORE2 algorithm demonstrated variable performance metrics for the different population cohorts. Overall, regarding diagnostic capacity, the AROC were 0.762 (0.752–0.772) and 0.718 (0.654–0.780) in Mexico and Portuguese cohorts, respectively (*Figure 3*). In all cases, the positive predictive values were below 7%, while the negative predictive values were all above 98%. Generally, the sensitivity properties of SCORE2 were high, 86% (Mexico) and 95% (Portugal), despite the lower specificity properties of 42.8% (Mexico) and 24.3% (Portugal), respectively.

**Table 3:** Diagnostic capacity, concordance, and calibration of the SCORE2 equation within each cohort

	Mexico	Portugal
**Diagnostic capacity**		
AROC (continuous)	0.762 (0.752–0.772)	0.718 (0.654–0.780)
Sensitivity^a^ (%)	85.7 (84.1–87.2)	95.0 (86.1–99.0)
Specificity^a^ (%)	42.8 (42.5–43.1)	24.3 (21.9–26.9)
Positive predictive value^a^ (%)	3.2 (3.0–3.3)	6.1 (4.7–7.9)
Negative predictive value^a^ (%)	99.3 (99.2–99.4)	98.9 (96.9–99.8)
**Concordance**		
Harrell’s C	0.717	0.692
**Calibration**		
Hosmer–Lemeshow test	<0.001	<0.001
**Overall predictive performance**		
Brier score	0.022	0.052

AROC, area under the receiver operating characteristic curve.

^a^A high to very high-risk category in detecting CVD events.

On the other hand, on assessing how well SCORE2 distinguished between participants with and without a subsequent CVD event 10-years after baseline risk assessment, the Harrell’s C statistic was 0.717 and 0.692 in the Mexican and in Portuguese cohorts, respectively.

**Figure 4:**
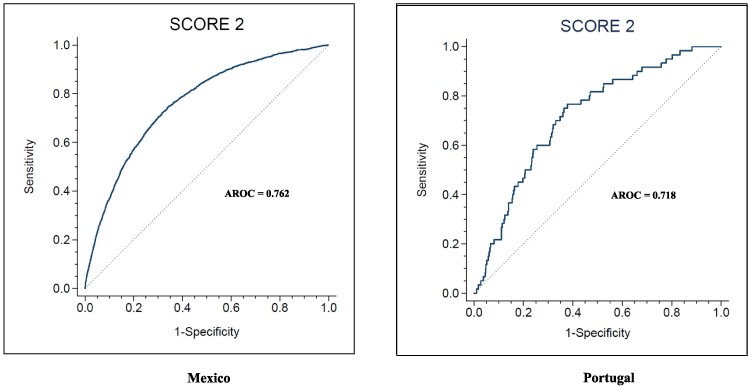
Calibration plots showing the agreement between observed and predicted probability for CVD, according to SCORE2 in each cohort.

Regarding the overall predictive performance of SCORE2, the Brier scores showed slightly different values of 0.022 in the Mexican cohort and 0.052 in the Portuguese cohort, respectively, suggesting reasonable predictive accuracy of the SCORE2 model in both populations. The calibration plots comparing the predicted and observed probabilities of the occurrence of CVD events in each cohort are shown in *Figure 4*. In Mexico, SCORE2 showed good calibration, especially in the low CVD-risk population group, with a slight overestimation in those with higher baseline CVD-risk. By comparison, in the Portuguese cohort, overestimation of the higher CVD risk categories was more substantial.

## Discussion

In this validation study, we comparatively assessed the relevance of the SCORE2 algorithm in predicting subsequent CVD risk 10-years later in two Hispanic populations, from Europe and Central America. The results suggest that SCORE2, originally validated in Western European populations, can be reasonably well applied in Mexican populations, whereas its use in Portuguese populations requires further refinement of the predictive parameters and testing in larger and more widely represented population samples. Albeit the Mexican cohort inherits ∼30% European ancestry,^12^ which might explain the positive findings, the general disparities observed between the two cohorts warrant further validation in larger samples that are country-representative. Although the Hosmer-Lemeshow test was significant in both cohorts, indicating miscalibration, similar findings have also been reported in other studies.^13,14^ Such findings could also be partly due to the sensitivity of the test to the sample size; for instance, the small size of the Portuguese cohort could lead to large differences between categories, while the large size of the Mexican cohort would lead to small differences being statistically significant.

### SCORE2 in the Portuguese cohort

CVD has been the leading cause of mortality in Europe, including Portugal, over the last four decades.^15^ Consequently, in 2023, the ESC updated the guidelines for CVD prevention, recommending the SCORE2 for estimating the 10-year risk of CVD events in European populations.^7,16^ According to SCORE2, all European countries were grouped into four risk regions based on WHO age- and sex-standardized absolute CVD rates; therefore, we classified Portugal as a moderate-risk region in this study to ensure consistency with the SCORE2 validation criteria. Although SCORE2 has been previously validated in Portugal, the study was limited to a cohort from Madeira,^17^ which is likely not fully representative of the continental Portuguese population. However, the AUROC obtained in the present analysis was similar to previously published findings, particularly for the percentage of low-to-moderate CVD risk. Conversely, the prevalence of individuals classified as being at a very high CVD risk was higher in the present analysis compared to the Madeira validation study. This could be due to differences in baseline characteristics, specifically, the predominantly male population with low diabetes and smoking prevalences in Madeira vs. our study population.^18^ The considerable overestimation of the predicted probabilities of the high CVD risk groups in Portuguese adults in our results suggests that while the SCORE2 predicted probabilities are well calibrated in the low-risk categories, their performance may vary more in higher-risk categories. Overall, our results suggest that SCORE2 might require recalibration to be fully operational in mainland Portugal.

### SCORE2 in the Mexican cohort

With the increasing trend in CVD mortality and morbidity globally, the burden of CVD is also increasing in Latin America,^13^ including Mexico.^19^ However, the CVD risk equations validated in Latin American populations remain limited, and there are substantial discrepancies in the performance of CVD risk-prediction models across several Latin American countries.^20,21^ To the best of our knowledge, this is the first study to validate SCORE2 in a Mexican cohort. According to our analysis in the Mexico City Prospective Study, SCORE2 had high predictive accuracy and good discrimination in the study population. Moreover, the application of SCORE2 in Mexicans showed good calibration, except for a slight overestimation in the higher CVD risk groups, suggesting that SCORE2 has the potential to work relatively well in Mexicans, at least in an urban setting. This might be due to the relatively high prevalence of diabetes in this population, which SCORE2 accounts for, and the high degree of inherited European ancestry. Therefore, it might be interesting to test whether the relatively acceptable performance of SCORE2 observed in this Mexican cohort extends to other Latin-American populations.

### Clinical implications

In both cohorts included, SCORE2 showed high sensitivity, a high negative predictive value, and moderate distinctive performance, supporting the SCORE2 equations as a good screening model for individuals with low CVD risk. However, the low specificity, low positive predictive value, and calibration performance with considerable overestimation in the higher CVD-risk groups indicate that SCORE2 could result in over-classifying individuals with high CVD risk, with potential implications for over-treatment. and psychological stress. The high sensitivity and low specificity in our findings suggest that only one in ten participants considered to be at risk for CVD subsequently developed CVD, while over 95% of participants classified as not being at risk for CVD did not develop CVD. within 10 years from the assessment of baseline risk. Therefore, the SCORE2 equation appears to correctly rule out the ‘true risk’ of CVD but needs further tuning for the correct detection of individuals who will really develop CVD among Mexican and Portuguese adults.

Nevertheless, it is worth noting that the SCORE2 algorithm accounts for geographic region, sex, age, smoking status, systolic blood pressure, and lipid profile, but does not account for the initiation of preventive treatments, such as statins or antihypertensive medications, during follow-up. Treatments can reduce the occurrence of CVD events, which may partly explain the observed overestimation of absolute CVD risk among high-risk groups in our analysis. Potential treatment effect effects are a common challenge in validation studies and highlight the complexity of assessment and recalibration of risk prediction algorithms in real-world settings where CVD-risk management is actively implemented. The discrimination and the overall predictive accuracy of the SCORE2 were moderate in both cohorts included in our analysis. Hence, as the ESC guidelines recommend, the interpretation of SCORE2 in decision making requires clinical judgement, particularly in patients taking lipid-lowering or antihypertensive treatments, patients with a family history of CVD, or chronic kidney disease, or patients exposed to low socioeconomic status.^7^ We therefore propose that although the use of alternative risk equations might be considered, they will always need to be validated for the target population before being applied in clinical practice.

### Strengths and limitations

The major strength of our study is the use of a standard methodology, which allows for direct comparison of the findings across two contemporaneous adult Hispanic populations with ancestry admixture. We employed high-quality-controlled laboratory values for lipids and renal function, carefully measured blood pressure, medical histories collected by trained fieldworkers, and medically certified CVD disease histories and causes of death. Our study also included under-represented populations from Central America and Europe, in whom SCORE2 has neither been validated before, nor tested for applicability. As our study suggests, SCORE2 (in its original form) has the potential to be used in contemporary Mexican adults, but it requires re-calibration.

Our study had several limitations. First, the ideal sample size for appropriate external validation requires a minimum of 200 CVD events.^22,23^ This threshold was met in the Mexico City Prospective Study but not in the EPIPorto study. This limits the statistical power in the Portuguese cohort and may lead to imprecise estimates of AUROC and the calibration coefficients. Therefore, the present findings should be interpreted with caution. Secondly, the methods for measuring blood pressure might differ, albeit standardization and within-study validity were performed within each cohort. Thirdly, there may be subtle differences in the methods used to register events attributed to cardiovascular diseases between countries. For instance, myocardial infarctions are commonly included in the Death Registry, while details for transient ischaemic attacks and stenting procedures are less specific. Fourthly, it was not possible to assess the status of participants who opted out after their last contact. As participants who opt out of prospective studies usually present with a higher prevalence of risk factors (i.e. smoking, diabetes) than those who remain in the cohort,^24,25^ it is likely that the incidence of events was underestimated compared to the predicted ones. This could partly explain the trend towards overestimation of the risk equations in both cohorts. Limitations also include a lack of generalizability to the whole country’s population since both cohorts consist of the urban population, with limited representation of the rural population of each country. CVD risk factors and the numbers of CVD events distribution can differ by socioeconomic and geographical contexts, and therefore, the performance of SCORE2 can vary accordingly. Additionally, calibration relies on mortality statistics from Western populations, which might differ from mortality rates in Latin America. Finally, the cohorts included in our analysis were mostly from an urban setting, and generalization to rural settings, although possible, might not be entirely transferable.

## Conclusion

SCORE2 performs reasonably well in assessing subsequent risk in a large Hispanic population from the Mexico City Prospective Study, supporting its suitability for use in clinical practice. In contrast, the performance of SCORE2 in the Portuguese cohort was less favourable, and the small number of events limits its validation. Further efforts should include the re-calibration in Portuguese and other Hispanic populations, before confirming the use of SCORE2 in clinical practice in such settings.

## Supplementary Material

qcag009_Supplementary_Data

## Data Availability

Data from the Mexico City Prospective Study cannot be shared publicly due to privacy or ethical restrictions. The data will be available to bona fide researchers upon request. For more details, the study’s Data and Sample Sharing policy may be downloaded (in English or Spanish) from https://www.ctsu.ox.ac.uk/research/mcps. Available study data can be examined in detail through the study’s Data Showcase, available at https://datashare.ndph.ox.ac.uk/mexico/. Data from the EPIPorto cohort cannot be shared publicly due to privacy or ethical restrictions. The data can be made available for research proposals upon request to the EPIPorto Executive Committee (epiporto@med.up.pt). Further information about the EPIPorto cohort can be obtained through this website: https://ispup.up.pt/coorte/epiporto/.
