# Supplementary material for: Validation of the European SCORE2 algorithm in one European and one Latin-American cohort studies with contrasting populations
Source: Eur Heart J Qual Care Clin Outcomes. 2026 Jan 27;12(5):863–71. doi: 10.1093/ehjqcco/qcag009 (PMC13457936; doi:10.1093/ehjqcco/qcag009)
Supplement: Supplementary file 1 [file qcag009_supplementary_data.zip › Sup tables_20250501.docx]

# SUPPLEMENTARY TABLES

**Supplementary Table** **1.1**: Details of cohorts used in the validation of SCORE2

| **No.** | **Study** | **Country** | **Initial number of participants** | **Average years of follow up** | **Paper describing the cohort (PMID)** |
| --- | --- | --- | --- | --- | --- |
| 1. | Mexico City Prospective Study | Mexico | 159,755 | 20.3 | 16556648 |
| 2. | EpiPorto | Portugal | 2,485 | 8.8 | 15246333 |

**Supplementary Table 1.2**: **Variables required to compute SCORE2.**

| **No.** | **Variables’ Names §** | **Explanation** |
| --- | --- | --- |
| 1. | ID | Research identifier |
| 2. | riskreg | Risk region (1= low risk, 2= medium risk, 3= high risk and 4= very high risk) ‡ |
| 3. | sex | Sex (1 = Male, 2 = Female) |
| 4. | ages | Age (years) |
| 5. | smallbin | Smoking status (0 = Other, 1 = Current) |
| 6. | hxdiabbin | History of diabetes (0 = No, 1 = Yes) |
| 7. | sbp | Systolic blood pressure (mm Hg) |
| 8. | tchol | Total cholesterol (mmol/L) |
| 9. | hdl | High-density lipoprotein cholesterol (mmol/L) |
| 10. | CVD_any | Any fatal or non-fatal CVD event (0=no, 1=yes) |
| 11 | FU_time | Time from baseline to first non-fatal CVD event, to death or stop of follow-up (years) |
| 12. | CVD_death | Any fatal CVD event (0=no, 1=yes) |
| 13. | Death | Any death from any medical cause (0=no, 1=yes) |
| 14. | FU_timed | Time from baseline to death or stop of follow-up (years) |

**§** as used in the statistical code**.** ‡ according to the ESC criteria, based on WHO mortality rates.

**Supplementary table 1.3**: **Variables required to re-calculation of diabetic patients or reassessing SCORE2**

| **No.** | **Variables’ Names §** | **Explanation** |
| --- | --- | --- |
| 1. | cr | Creatinine (units) to compute glomerular filtration rate (in μmol) as per CKD epi equation |
| 2. | antihta | Any antihypertensive drug treatment (0=no, 1=yes) |
| 3. | hta | Hypertension, defined as SBP≥140 mmHg or DBP≥90 mmHg or antihypertensive drug treatment |
| 4. | lip | Any hypolipidemic drug treatment (0=no, 1=yes) |
| 5. | trig | Triglycerides, fasting (mmol/l) |
| 6. | ldlch | LDL cholesterol (mmol/l) |
| 7. | ethnicity | 1=black, 0=other |

**§** as used in the statistical code**.**
